# Supplementary figures and images for: Production of a functionally active recombinant SARS-CoV-2 (COVID-19) 3C-like protease and a soluble inactive 3C-like protease-RBD chimeric in a prokaryotic expression system
Source: Epidemiol Infect. 2022 Jun 20;150:e128. doi: 10.1017/S0950268822001078 (PMC9300977; doi:10.1017/S0950268822001078)

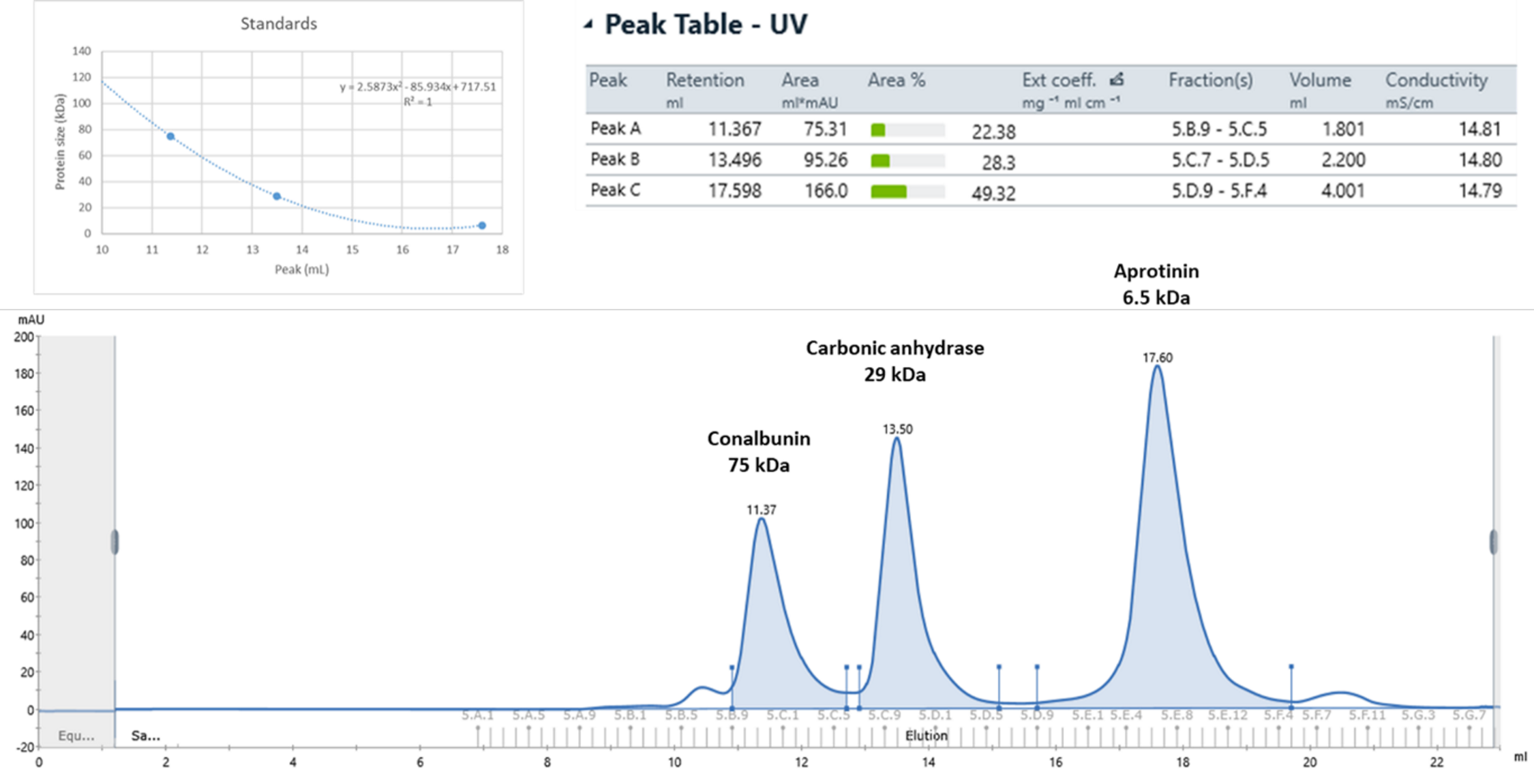

Supplement: Supplementary file 1 [file hygsup.zip › S0950268822001078sup001.tif]

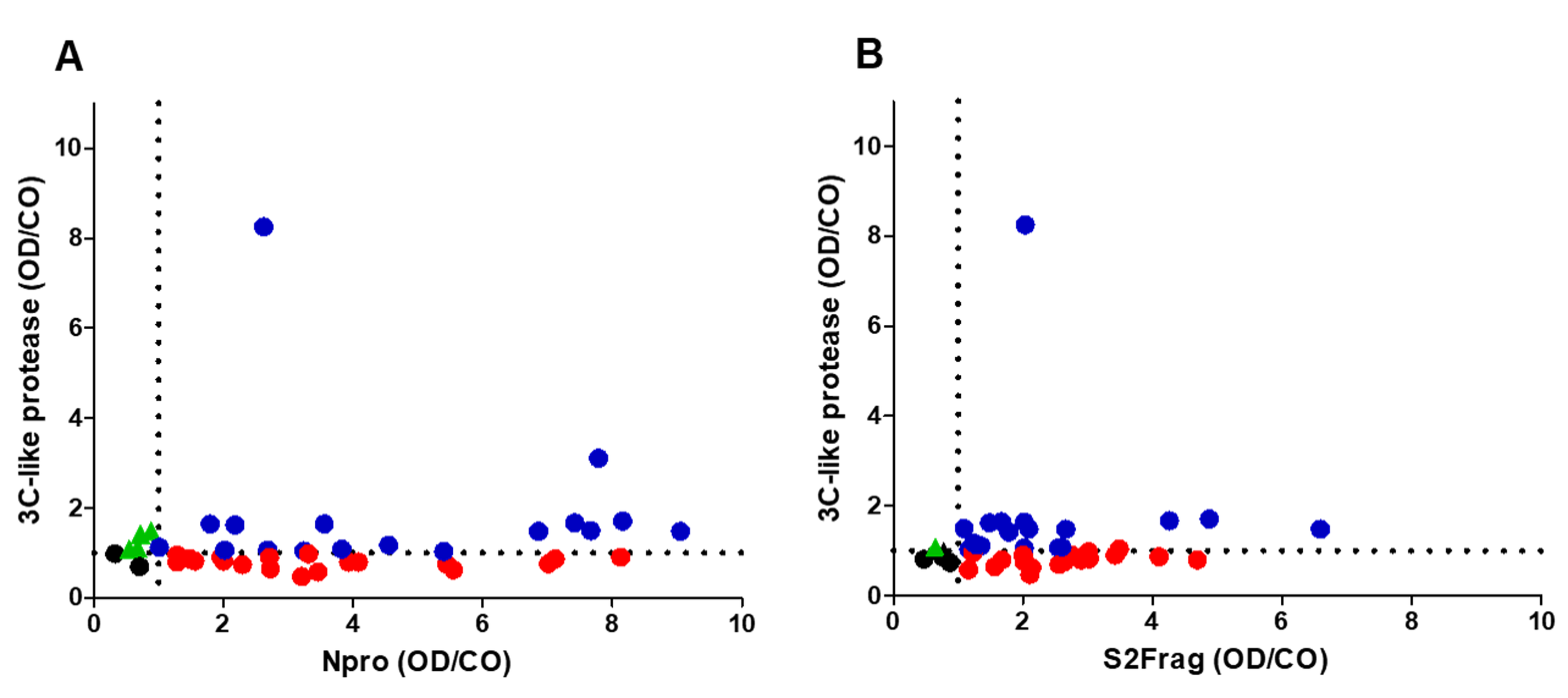

Supplement: Supplementary file 1 [file hygsup.zip › S0950268822001078sup002.tif]
